# Lack of *Wdr13* Gene in Mice Leads to Enhanced Pancreatic Beta Cell Proliferation, Hyperinsulinemia and Mild Obesity

**DOI:** 10.1371/journal.pone.0038685

**Published:** 2012-06-08

**Authors:** Vijay Pratap Singh, B. Jyothi Lakshmi, Shalu Singh, Vanya Shah, Sandeep Goel, D. Partha Sarathi, Satish Kumar

**Affiliations:** Centre for Cellular and Molecular Biology, Council of Scientific and Industrial Research (CSIR), Hyderabad, India; Montreal Diabetes Research Center, Canada

## Abstract

WD-repeat proteins are very diverse, yet these are structurally related proteins that participate in a wide range of cellular functions. WDR13, a member of this family, is conserved from fishes to humans and localizes into the nucleus. To understand the in vivo function(s) of *Wdr13* gene, we have created and characterized a mutant mouse strain lacking this gene. The mutant mice had higher serum insulin levels and increased pancreatic islet mass as a result of enhanced beta cell proliferation. While a known cell cycle inhibitor, p21, was downregulated in the mutant islets, over expression of WDR13 in the pancreatic beta cell line (MIN6) resulted in upregulation of p21, accompanied by retardation of cell proliferation. We suggest that WDR13 is a novel negative regulator of the pancreatic beta cell proliferation. Given the higher insulin levels and better glucose clearance in *Wdr13* gene deficient mice, we propose that this protein may be a potential candidate drug target for ameliorating impaired glucose metabolism in diabetes.

## Introduction

WD (tryptophan and aspartate)-repeat proteins belong to a large family of structurally related proteins, members of which have diverse functions such as cell cycle regulation, transcription, chromatin organization and protein trafficking [1], [2]. These proteins provide a platform for protein-protein interactions. WDR13 protein is a member of this family and localizes to the nucleus [3]. *Wdr13* gene is highly conserved in vertebrates. This gene is located on the X-chromosome at locus Xp11.23 and XA1.1 in human and mouse, respectively. In human, X chromosomal deletions including this gene have been associated with mental retardation, obesity and xeroderma [4], [5], [6], [7]. *Wdr13* gene expresses in most of the tissues [8], the highest level of expression being in pancreas, brain, testis and ovaries. Several WD-repeat proteins have been identified that are expressed in pancreatic beta cells and have roles in beta cell proliferation [9], [10].

The beta cell mass is regulated by the balance between neogenesis/proliferation and apoptosis/necrosis. In mice, differentiation of islet precursor and expansion are responsible for beta cell neogenesis until the first week of life [11], [12]. Thereafter, expansion of existing beta cells is the main source of newly formed beta cells [13], [14]. In pathological conditions there can be alpha to beta cell trans-differentiation [15]. Various cell cycle regulators have been identified that have role in pancreatic beta cell proliferation [16]. Cell cycle progression in pancreatic islet is controlled by cyclins, cyclin dependent kinases (CDKs), cyclin dependent kinase inhibitors and hormones [16], [17].

Pancreatic islet mass, insulin production and body weight are inter-related [12], [18]. Insulin levels have been positively correlated with obesity in humans [19] and rodents [20]. Generally, obesity leads to higher demand for insulin production and the same is met by increase in beta cell mass. Obesity is also a major risk factor for the onset of peripheral insulin resistance [21]. Insulin resistance leads to further higher demand for insulin from beta cells, and eventually triggering beta cell failure. This results in beta cell survival defects, insufficient beta cell mass and deterioration of the key beta cell function i.e. glucose stimulated insulin secretion, and ultimately type 2 diabetes. Thus, the mass of insulin producing beta cells changes dynamically according to the metabolic conditions [22], [23]. Alternatively, obesity may be a consequence of higher insulin levels [24], [25], [26] as insulin has stimulatory effect on adipogenesis by increasing the lipid accumulation in adipocytes [27], [28], [29]. Insulin is also involved in adipocyte survival [30]. Adipose tissue-specific insulin receptor gene knockout protects against obesity, emphasizing that insulin signaling to adipocytes is important for development of obesity [28]. Hyper insulin secretion in MOR-1 opioid receptor knockout mice results in higher body weight [26] whereas CHOP knockout mice become obese with increasing insulin secretion although glucose tolerance remains unchanged in these mice [31].

To understand the in vivo role of *Wdr13* gene, we have created a mouse strain lacking this gene and show that these mice have higher pancreatic islet mass as a result of higher beta cell proliferation, develop hyperinsulinemia and mild obesity.

## Results

### Generation of *Wdr13* knockout mice

**Figure 1:**
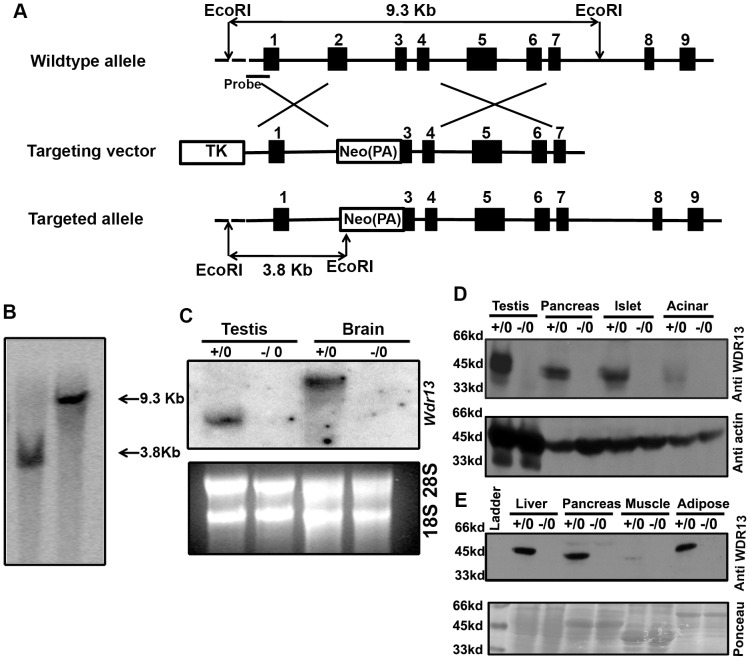
Generation of *Wdr*13 knockout mice. A) Exon2 and exon3 (partial) of *Wdr*13 gene were replaced by neomycin resistance marker gene. B) Southern blot analysis showing 9.3 kb *Eco*RI fragment from the wild type allele and 3.8 kb fragment from the mutant allele using a 700 bp probe from 5′ end of the locus. C) Northern blot analysis by *Wdr*13 cDNA probe showing the absence of *Wdr*13 transcript in *Wdr*13−/0 mice (upper panel) and ethidium bromide staining as loading control (lower panel). D) Western blot analysis from testis, pancreas and purified islets using anti-WDR13 rabbit polyclonal antibody (upper panel) showing the absence of WDR13 protein in *Wdr*13−/0 mice, and anti-beta actin as loading control (lower panel). The expression of WDR13 is extremely high in islets in comparison to acinar cells. E) Expression of WDR13 protein in insulin sensitive tissues such as liver, pancreas, muscle and adipose tissues using anti WDR13 antibody (upper panel) and posceau stained blot as loading control (lower panel). (+/0 wild type male; −/0 knockout male).

*Wdr13* is a single copy gene located on the X-chromosome [8]. The targeting strategy was designed to substitute exon 2, intron 2 and exon 3 (partial) of the endogenous gene with neomycin gene cassette containing polyA (Figure 1A). Targeted ES cell clones were identified by southern blot (Figure 1B). Chimaeras were generated from one of the targeted ES cell clones after injecting these cells into C57BL/6 blastocysts. Male chimaeras were bred with CD-1 females and germ line transmission from the ES cell component was identified by the presence of agouti progeny. The mutant allele in these progeny was further confirmed by southern blot. Northern blot analysis using *Wdr13* cDNA probe revealed the lack of 4 kb and 2 kb transcripts from brain and testis of the knockout mice, respectively as compared to that from the wild type mice (Figure 1C). Further, western blot using anti WDR13 antibody showed the absence of WDR13 protein in various tissues of the knockout mice (Figure 1D, E). These results confirmed that targeting of *Wdr13* gene had led to the generation of a null allele.

### *Wdr13* knockout mice were viable and fertile

*Wdr13*−/0 male and *Wdr13*−/− female knockout mice were viable and fertile. Given the comparatively high level of expression of *Wdr13* gene in spermatogonia and spermatocytes, and the presence of a different size transcript in testis [8], we analysed the litter size from various matings involving the mutant and wild type mice. The genotypes of the parents had no effects on the litter size (Table S1). The sperm number from *Wdr13*−/0 males (40.6±5.49 million/ml) was similar to that of *Wdr13*+/0 (37.9±3.05 million/ml).

### Increased body weight of *Wdr13* knockout mice

**Figure 2:**
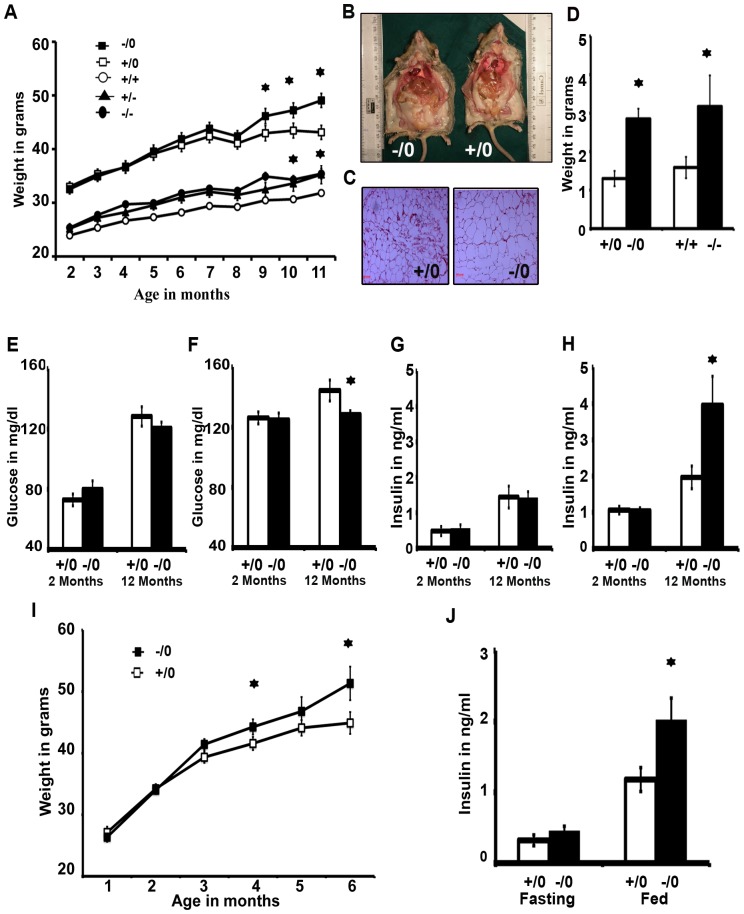
Body weight, body composition, and glucose and insulin levels of *Wdr*13 knockout mice fed on normal chow and high fat diet. A) Growth curve of *Wdr*13+/0 male, *Wdr*13−/0 male, *Wdr*13+/+ female, *Wdr*13+/− female and *Wdr*13−/− female (n = 12) on normal chow. B) Increased adipose tissue mass in *Wdr*13−/0 mice. C) H&E staining of a section of epididymal fat pad of *Wdr*13−/0 mouse showing hypertrophy of adipose tissues D) Weight of epididymal fat pad in male and ovarian fat pad in female at 12 months (n = 6) of the mutant and wild type mice. E) Sixteen hours fasting glucose level in *Wdr*13 knockout mice at 2 months and 12 months. F) Random fed glucose level in *Wdr*13 knockout mice at 2 months and 12 months G) Fasting insulin level in *Wdr*13 knockout mice at 2 months and 12 months H) Random fed insulin level in *Wdr*13 knockout mice at 2 months and 12 months. I) Growth curve of *Wdr*13+/0 male and *Wdr*13−/0 male on high fat diet (n = 10 to12). J) Fasting and fed insulin level in *Wdr*13−/0 mice and in their wild type littermates at 6 months. (+/0 wild type male; −/0 knockout male; +/+ wild type female; +/− heterozygous female; −/− knockout female).

*Wdr13* deficient mice differentiated in body weight from their wild type littermates around nine months of age when fed normal chow. At 11 months the mutant male and female mice had 13% and 11% higher body weights (P≤0.05), respectively than their littermates (Figure 2A, B). The weight of epididymal fat pad was 2.5-fold more in *Wdr13*−/0 mice in comparison to that of wild type, whereas the weight of ovarian fat pad was 2- fold more in *Wdr13*−/− in comparison to that of the wild type (Figure 2D). Histological examination of epididymal fat pad revealed adipocyte hypertrophy in *Wdr13* knockout mice (Figure 2C). Adipocyte hypertrophy was also noticed in skin sections of the mutant mice. Various organs including, brain, lung, heart, liver, pancreas, spleen and testis of the mutant and wild type mice were weighed at 12 months. The mutant mice had significantly higher pancreatic weight. There was no difference in the weights of other organs (Table S2). To understand the effect of diet on the higher body weight of the mutant mice, one month old mice were kept on a high fat (60%) diet and their body weights were followed. Interestingly, *Wdr13*−/0 weighed significantly more at five months (Figure 2I) indicating the advancement of the obesity phenotype of the mutant mice.

### Glucose and insulin levels in *Wdr13* knockout mice

Given the age-dependent obesity phenotype, we measured fasting and random blood glucose levels at different age points in mice fed with normal chow (Figure 2E, F). There was no effect of *Wdr13* genotype on fasting glucose levels. Similarly, the random glucose levels at 2 months did not differ between the mutant and wild type mice. However, the mutant mice showed lower random glucose at 12 months on normal chow. In the light of lower random glucose at 12 months, we estimated the serum insulin levels. The fasting insulin levels were similar in the wild type and mutant mice at both age points (Figure 2G). While random insulin levels in *Wdr13* deficient and wild type mice at 2 months were similar, interestingly, at 12 months the mutant mice had 2.13 fold more random insulin level on normal chow (Figure 2H). Similarly, the mutant mice had 1.6-fold more random insulin levels at six months when fed on a high fat diet (Figure 2J).

### Increased glucose clearance but unaltered insulin sensitivity in *Wdr13* knockout mice

**Figure 3:**
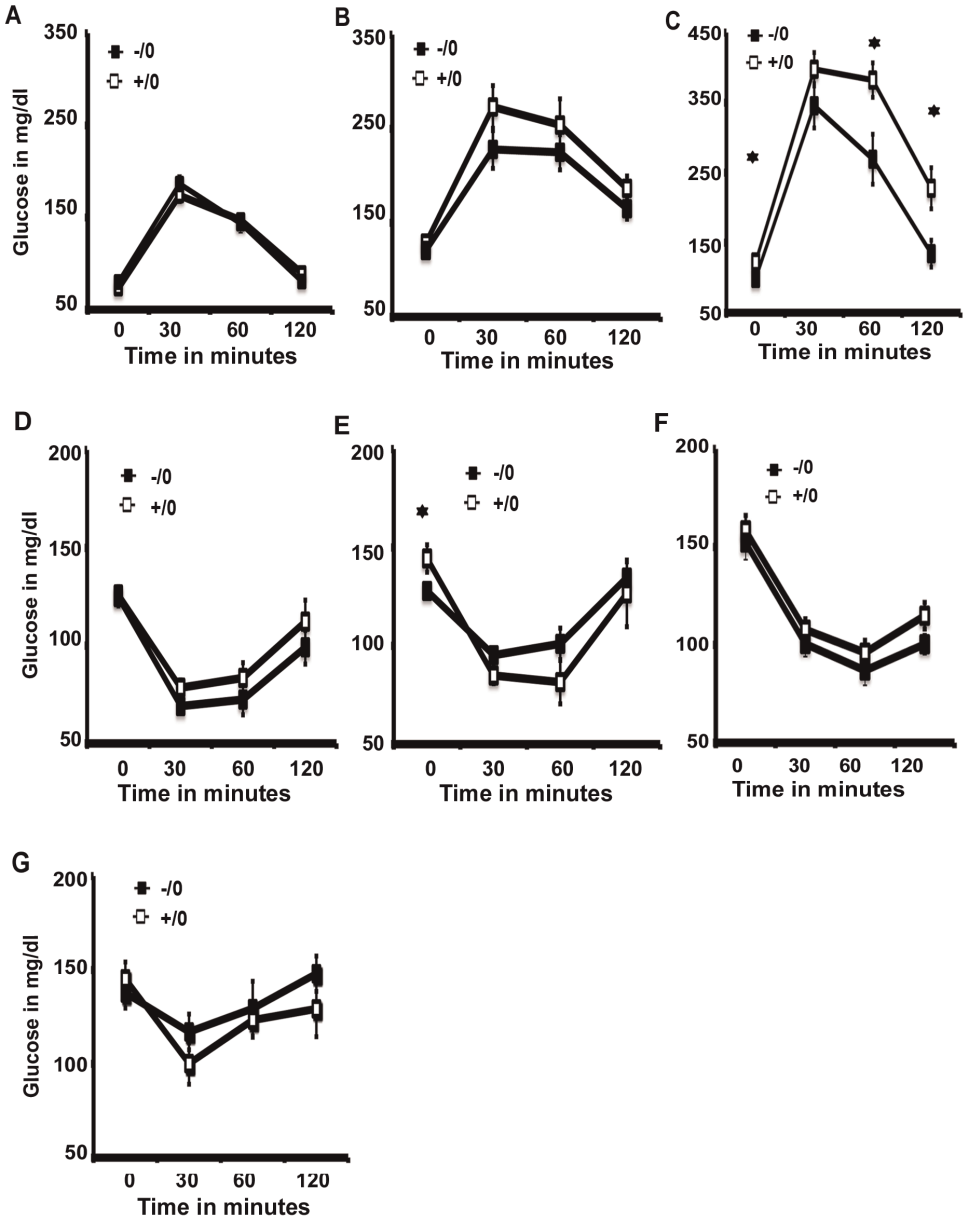
Glucose tolerance test (GTT) and Insulin tolerance test (ITT) of *Wdr13* knockout mice. A) GTT at 2 months on normal chow (n = 8–12). B) GTT at 12 months on normal chow (n = 8–10). C) Glucose tolerance test at 6 months on high fat diet (n = 8). D) ITT at 2 months on normal chow (n = 8–12). E) ITT at 12 months on normal chow (n = 8–10). F) Insulin tolerance test at 6 months on high fat diet (n = 8) G) ITT at 9 months on high fat diet (n = 6–8). (+/0 wild type male; −/0 knockout male).

Given the high level of random insulin in *Wdr13* deficient mice, we challenged these mice with glucose to determine their glucose clearance at 2 months and 12 months on normal chow, and at 6 months on a high fat diet. The mutant and wild type mice showed similar glucose clearance (Figure 3A) at 2 months. At 12 months the mutant mice appeared to have marginally better glucose clearance; however, the difference was statistically non-significant (Figure 3B). However, the mutant mice showed consistently better glucose clearance at 6 months on a high fat diet (Figure 3C). Insulin tolerance test (ITT) at 2 months (Figure 3D) and 12 months (Figure 3E) did not reveal any difference between the mutant and wild type mice fed on normal chow, indicating that insulin sensitivity was similar in these two groups. Consistent with this, insulin sensitivity in the mutant mice at 6 months (Figure 3F) and 9 months (Figure 3G) was similar to that of wild type mice when fed on a high fat diet.

### Increased islet mass and beta cell proliferation in *Wdr13* knockout mice

**Figure 4:**
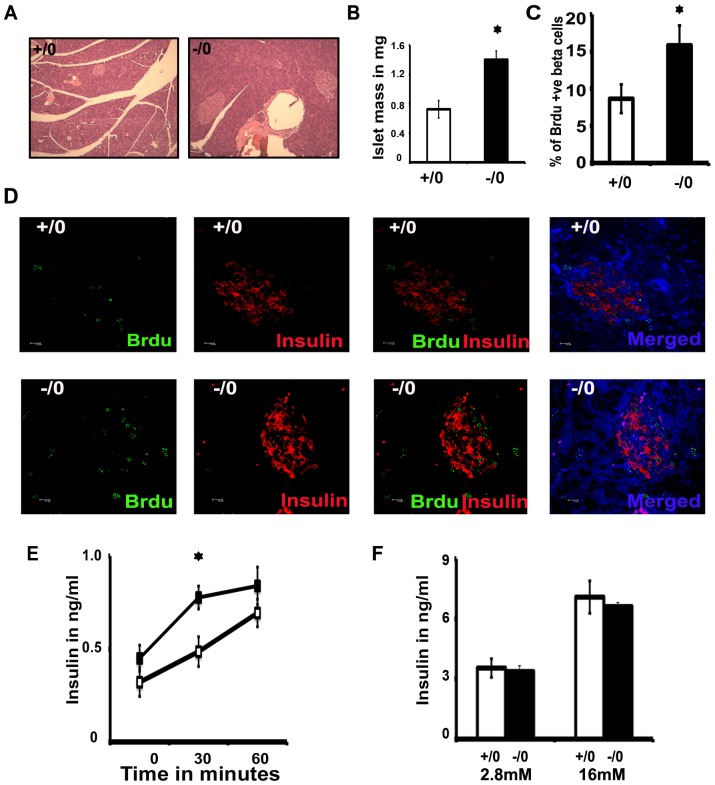
Histology of pancreatic islet, pancreatic beta cell proliferation, in-vivo and in-vitro insulin secretion in *Wdr*13 knockout mice. A) Islet morphology showing increased islet mass by H&E staining. B) Islet mass in mg at 6 months on high fat diet showing increased total islet mass in *Wdr*13 knockout mice (n = 4). C) Percent of dividing cells in islets by BrdU labeling (n = 309–517). D) Analysis of pancreatic beta cells proliferation analysis by BrdU labeling showing more proliferating beta cells in *Wdr13* knockout islets. E) In-vivo insulin secretion in response to glucose at 6 months (n = 4 to 6). F) In-vitro insulin secretion from isolated pancreatic islet of the wild type and *Wdr*13 knockout mice at 2.8 mM or 16 mM glucose concentration (+/0 wild type male; −/0 knockout male).

Since *Wdr13* deficient mice were hyperinsulinemic and mildly obese, we analyzed the pancreatic histology at 6 months in these mice when fed on a high fat diet. Interestingly, total islet mass was significantly more in *Wdr13* knockout mice at 6 months (Figure 4A, B). To understand the increased islet mass, we measured beta cell proliferation by in-situ BrdU labeling. Briefly, one month old mice were kept on a high fat diet for three weeks before infusion of BrdU and we observed a 2-fold more proliferation of beta cells in *Wdr13* mutant mice as compared to their wild type littermates (Figure 4C, D).

### Higher insulin level in knockout mice is due to the increased islet mass rather than insulin secretion per unit islet

Given the better glucose clearance in *Wdr13*−/0 mice, glucose was injected into 6 month old (on high fat diet) fasting mice and the insulin secretion was monitored at different time points (Figure 4E). Insulin secretion after 30 minutes of glucose injection was 1.8 fold more in *Wdr13*−/0 mice. The higher insulin levels in the mutant mice might result from the increased islet mass observed by us in these mice (Figure 4A, B) or might from higher insulin secretion per unit beta cell in response to glucose stimulation. To rule out the latter possibility, we isolated islets from 3 month old *Wdr13*−/0 mice and their wild type littermates (n = 3). Twenty-five equal sized islets from each mouse were induced with 16 mM glucose and insulin secretion was measured after 60 minutes. There was no difference in the in vitro insulin secretion between the mutant and wild type islets (Figure 4F).

### Overexpression of *Wdr13* gene results in cell growth retardation

**Figure 5:**
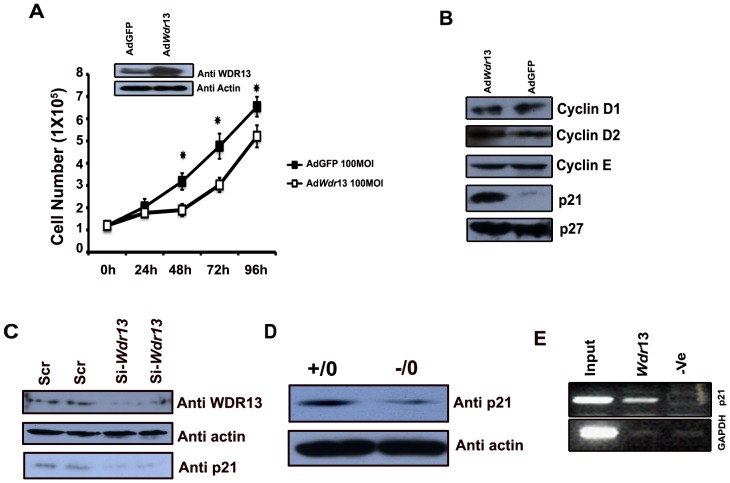
Effect of WRD13 levels on pancreatic beta cell proliferation and cell cycle regulators. A) Transfection with Ad*Wdr*13 and AdGFP viruses shows overexpression of WDR13 protein in MIN6 cells as visualized by immunoblotting using anti WDR13 antibody. Lower panel shows beta actin as loading control. Overexpression of WDR13 protein results in retardation in cell proliferation after 48 h of transfection with 100 MOI. B) Overexpression of WDR13 protein results in accumulation of p21, whereas Cyclin D1, Cyclin D2, Cyclin E1 and p27 levels remain unaffected. C) siRNA knockdown of WDR13 in MIN6 cells. MIN6 cells were transfected with nonspecific scrambled (Scr) siRNAs and WDR13 specific siRNA. Immunoblot analysis shows Knockdown of WDR13 protein. Actin was used as loading control. Immunoblot, using p21 antibody shows reduction of p21 levels in WDR13 knockdown MIN6 cell. D) Cell cycle inhibitor p21 expression in purified pancreatic islets of *Wdr*13 knockout mice and that of wild type littermates by western blot analysis. Beta actin was used as loading control. p21 expression is less in the islets of knockout mice. E) Occupancy by WDR13 at p21 promoter revealed by chromatin immunoprecipitation using primers specific for p21 and GAPDH.

To further understand the role of *Wdr13* gene in beta cell proliferation, WDR13 protein was overexpressed in MIN6 cell line using pAd Easy system. MIN6 cells were transfected with AdGFP and Ad*Wdr13* with 100 MOI each in 24 well plates. After 48 hours of transfection, overexpression of *Wdr13* gene was confirmed by western blot (Figure 5A) using anti WDR13 antibody. Further, we studied the growth curve of MIN6 cells by counting cells after overexpression of *Wdr13* gene. A significant reduction in cell growth was observed 48 h post transfection (Figure 5A).

### Effect of WDR13 on cell cycle regulators

To understand the growth retardation after overexpression of WDR13, various cell cycle regulators were analyzed by western blotting. The protein levels of cyclin D1, cyclin D2, cyclin E and p27 were unaltered, whereas p21 was highly upregulated (Figure 5B). Conversely, siRNA knockdown of *Wdr13* gene showed downregulation of p21 protein levels (Figure 5C). Further, consistent with these results western blot analysis revealed reduction in p21 levels in the islets from *Wdr13* knockout mice, (Figure 5D). To understand the nature of p21 regulation by WDR13, promoter occupancy of WDR13 protein was analyzed at p21 promoter. Chromatin immunoprecipitation experiment showed interaction of WDR13 with p21 promoter sequences indicating a role of WDR13 in p21 regulation (Figure 5E).

## Discussion

In the present study, we have examined the in vivo role of one of the WD repeat proteins, namely, WDR13 by knocking out this gene in mouse. The mutant mice were viable and fertile without any overt phenotype except that the mice were significantly heavier than their wild type littermates at around nine months and continued to weigh more till the termination of the growth experiment at 12 months when fed on normal chow. This age-dependent higher body weight of the mutant mice advanced to five months when these mice were fed on a high fat diet (Figure 2I). Anatomical and histological examination revealed that the increased body weights of the mutant mice were mainly due to the increase in the adipose tissue volume/weight resulting from hypertrophy of adipocytes without any indication of change in adipocyte numbers (Figure 2C).

*Wdr13* knockout mice were mildly obese, and random insulin levels in these mice were ∼2 fold higher as compared to their wild type littermates at 12 months on normal chow and at 6 months on a high fat diet. However, there was no difference in fasting insulin levels. Dynamic changes in the insulin-producing pancreatic beta cell mass are dependent on metabolic conditions, and positive correlations between body weight, insulin production and islet mass are well documented [12], [32]. Various studies have shown that the increase in insulin levels may be a compensatory mechanism to the decreased peripheral insulin sensitivity in response to obesity, ultimately leading to islet failure and to type 2 diabetes [33]. On the other hand, it is also known that higher insulin levels result in higher glucose uptake by adipose tissues, which would in turn alter the lipid metabolism and adipogenesis [34]. Consistent with the latter findings, insulin receptor knockout mice exhibited decreased adipose tissue [35]. Insulin stimulates hepatic lipogenesis as well as fatty acid uptake in adipocytes leading to increased adipose tissue formation [27]. Moreover, insulin receptor glucose transporter-4 pathway helps to convert glucose to lipid in adipose tissues [34]. In addition to the role of insulin in adipogenesis, insulin secretion has been positively correlated with obesity in humans [19], rodents [20] and non mammalian avian models [36]. In *Wdr13* knockout mice, islet mass, insulin levels and glucose-stimulated insulin secretion were more at 6 months on a high fat diet. Notwithstanding higher body weights at 6 month and onwards, the mutant mice had better glucose clearance, without any indication of diabetes till the termination of experiments at 9 months of age in the case of a high fat diet and 12 months on normal chow (Figure 3). Further, ITT experiments showed similar insulin sensitivity in *Wdr13* knockout mice as their wild type littermates. However, the lack of insulin resistance as revealed by ITT data would require confirmation using hyperinsulinemic clamp studies. Nevertheless, it appears that the body weight gain in *Wdr13* knockout mice may be a consequence of insulin hypersecretion, which in turn may alter adipogenesis in fat cells. Hyperinsulinemia, accompanied by mild obesity in *Wdr13* knockout mice appears to be reminiscent of MOR1 [26] and chop [25], [31] knockout mice, where adiposity is enhanced by higher insulin secretion. It may be recalled that *Wdr13* gene is expressed at high levels in brain and at moderate levels in liver and adipose tissues (Figure 1). Therefore, we cannot rule out the possibility that the origin of obesity in our mutant mice reflected the deficiency of this protein in any one or all of these tissues. In this context, it may be noted that the food intake in *Wdr13* knockout mice was marginally higher than that in wild type littermates (Figure S1); the difference was statistically non-significant. Although our in-vitro data in MIN6 cells as well as hyperinsulinemia and better glucose clearance in *Wdr13* knockout mice show functional significance of WDR13 protein in pancreatic beta cells, a tissue-specific knockout of *Wdr13* gene will be necessary to confirm that the gross changes observed by us in the mutant mice are direct consequences of alterations in beta cell function in these mice.

We have earlier reported relatively higher level of expression of *Wdr13* in pancreas [3], and the expression of this gene in the pancreatic islets is much more as compared to extremely low levels seen in the acinar cells (Figure 1D). *Wdr13* knockout mice had more pancreatic islet mass than their littermates when fed on a high fat diet for five months after weaning at one month. To understand the phenomenon of the increased islet mass, we assayed in vivo beta cell proliferation after one-month old mice were fed on a high fat diet for three weeks. *Wdr13* knockout mice had enhanced beta cell proliferation as compared to that in their wild type littermates (Figure 4C, D). We have not analyzed the proliferation rates of other cell types in the pancreatic islets. Given the fact that beta cells contribute to 80–85% mass of pancreatic islets, it would appear that the increase in islet mass in *Wdr13* knockout mice might be primarily due to the enhanced beta cell proliferation. This conclusion is further strengthened by reversal of the cell proliferation phenotype i.e. the growth retardation observed by us in the pancreatic MIN6 cells upon overexpression of *Wdr13* gene (Figure 5A). The lack of difference between in vitro insulin secretion capacities of the wild type and knockout pancreatic islets (Figure 4F) provides further indirect support to the above conclusion. Various extrinsic and intrinsic factors responsible for beta cell proliferation have been reported [12], [16]. Many positive regulators of beta cells exist which include incretins [37], EGF [38], lactogens and growth hormones [39], HNF-4a [40], calcineurin/NFAT [41], Wnt3a [42] and integrins [43]. Various cell cycle inhibitors (p15, p16, p18, p19, p21, p27 and p57) have been identified, which target either various cyclins or cyclin dependent kinases to inhibit progression at various stages of the cell cycle [16]. In the present study, overexpression of WDR13 in pancreatic MIN6 cell line resulted in significantly higher p21 protein level, while cyclin D1, cyclin D2, cyclin E and p27 levels remained unaltered (Figure 5B). Further, through ChIP assay, we have identified occupancy of p21 promoter by WDR13 in pancreatic MIN6 cells. Consistent with these results, knockdown of WDR13 using siRNA in MIN6 cells and *Wdr13* knockout pancreatic islets showed downregulation of p21 protein (Figure 5C, D). Cozar-Castellano *et al.*, [44] observed an inhibitory role of p21 on beta cell proliferation in mice transgenic for hepatocyte growth factor and placental lactogen. However, in another study this group showed that knockout of p21 did not affect beta cell proliferation [45]. It might be possible that other protein(s) compensated the complete loss of p21 in beta cells in the latter study.

WD-repeat proteins provide a platform for protein-protein interactions [1]. WDR13 protein contains five putative WD repeats and a LxxLL motif at c-terminal. It is possible that WDR13 mediates its effect (s) by interacting with other proteins. The effect of WDR13 on p21 levels observed by us in the present study may be the result of either direct or indirect interactions of this protein with p21 promoter. However, no DNA binding domain has been predicted in WDR13 [8]. Therefore, it is probable that WDR13 may either be activating p21 promoter by interacting with other proteins or repressing a repressor of p21. Our results will be useful in further unraveling the mechanisms of beta cell cycle regulation. It may be recalled that *Wdr13* gene is expressed in most of the tissues and some of the interactions of this protein with its partners may even turn out to be tissue-specific; consequently, the precise mechanism(s) of action may differ in different tissues and/or at different target genes. Extensive CHIP experiments will be necessary to unravel different target genes of WDR13 and to understand the mode of action of this protein at these loci in various cell types.

In conclusion, we provide evidence that WDR13 deficiency in mice leads to increased pancreatic islet mass, hyperinsulinemia, better glucose clearance and mild obesity. Our present study reveals a role of WDR13 in regulation of beta cell proliferation and these results provide a basis for further investigations aimed at delineating the mechanisms of action of WDR13 in cell cycle regulation through p21 and other as yet unidentified targets of this protein. We propose that given the higher random insulin levels and better glucose clearance in *Wdr13* knockout mice, this protein may be explored as a potential candidate drug target for ameliorating impaired glucose metabolism in diabetes.

## Materials and Methods

### Ethics statement

The institutional Animal Ethics Committee of the Centre for Cellular and Molecular Biology, Hyderabad, India approved all the mice experiments.

### Generation of *Wdr13* null mice

To construct the *Wdr13* gene targeting vector, a 7.1 kb *Hind*III fragment from this gene including exon1 to exon7 was sub-cloned in pBluescript II KS vector (Stratagene). A 1.35 kb region from this fragment spanning exon 2 and exon 3 (partial) was replaced by *Xho*I-*Sal*I fragment of pMC1neo Poly A (Stratagene). To further enrich for the targeting events, a negatively selectable HSV-tk gene was placed before the 5′ end of the homologous sequences from *Wdr13* gene. The resulting vector had homologies of 1.6 kb and 4.1 kb at the 5′ and 3′ends, respectively. Forty micrograms of linearized targeting vector DNA was electroporated into R1 ES cells [46]. The ES cells were selected with G418 (0.25 mg/ml) and ganciclovir (2 µM). To identify the targeted clones, genomic DNA was isolated from ES cells and southern hybridization was performed using a 700 bp *Eco*RV-*Bam*HI fragment as a probe from the 5′ end of this locus. One of the targeted clones was injected into 3.5-dpc C57BL/6 blastocysts and the latter were transferred into the uteri of CD1 pseudopregnant females. To obtain germline transmission of the mutant allele, chimaeric male mice were mated with CD1 females. Germ line transmission was confirmed by southern analysis. PCR was used for regular screening of mutant mice using a primer pair (Table S3) from the *Wdr13* locus and another primer pair from neomycin gene.

### Northern blot and western blot analysis

Various tissues were snap frozen in liquid nitrogen and stored at −80°C till further use. Total RNA was isolated using RNeasy Mini Kit (Qiagen). For Northern analysis, RNA was electrophoresed on 1% agarose gel containing 2.2 M formaldehyde and blotted on hybond N+ membrane with 50 mM NaOH. *Wdr13* cDNA was radio labeled with αdATP using random priming kit. Hybridization was performed overnight at 65°C in 0.5 M phosphate buffer/7% SDS/1 mM EDTA and the membranes were then washed 3× at 65°C in 40 mM phosphate buffer/1% SDS/1 mM EDTA. The membranes were exposed to X-ray sheets (Fuji films) and developed. Proteins were extracted from various tissues, separated on 10% SDS-PAGE, blotted on PVDF membranes and western blots were performed. Anti-WDR13 antibody (HPA000913) from Sigma and p21 (sc-397), p27 (sc-528), cyclinD1 (sc-246), cyclinD2 (sc-593) and cyclinE (sc-481) from Santacruz were used for visualization of the respective protein.

### Animal weight measurement, feed consumption and body composition

Mice were housed in temperature, humidity and light/dark cycle (12 hrs 6am- 6pm) controlled animal rooms. Autoclaved normal diet or high fat diet was fed *ad libitum*, and the feed intake was measured weekly. Mice were weighed fortnightly. To determine the body composition, mice were dissected and the weights of various organs were measured.

### GTT, ITT, and insulin measurements

Blood glucose was measured with a glucometer using Sensor Comfort strips (Accu-Check) and insulin was measured by ELISA kits (Linco Research). To compare glucose clearance and insulin secretion, the mice were kept off-feed for 16 hours, glucose was injected intraperitoneally (2.0 grams glucose/Kg body weight) and blood was collected at 0, 30, 60 and 120 minutes intervals. Insulin tolerance was estimated by measuring glucose levels at 0, 30, 60 and 120 minutes intervals after injecting human insulin intraperitoneally (1 U/kg).

### Islet isolation and in-vitro insulin secretion

Pancreatic islets were isolated from the wild type and knockout mice as described by Wen *et al.* [26]. Briefly, 2 mg/ml collagenase type IV (Invitrogen) dissolved in Hanks' balanced salt solution (HBSS) was injected into common bile duct and pancreas were incubated at 37°C for 30 minutes. After collagenase digestion, pancreatic tissue was washed twice with cold HBSS and islets were purified by Ficoll gradient. The purified islets were washed two times with HBSS and transferred to RPMI1640 medium supplemented with 10% fetal bovine serum 100 U/ml penicillin, and 50 µg/ml streptomycin for overnight recovery. The islets were transferred next morning to Krebs–Ringer bicarbonate buffer (111 mM NaCl, 4.8 mM KCl, 2.3 mM CaCl_2_, 1.2 mM MgSO_4_, 25 mM NaHCO_3_) supplemented with 10 mM HEES, 2.8 mM glucose, 0.2% BSA and incubated for 90 minutes at 37°C with 5% CO_2_. To measure the insulin secretion in vitro, five equal size islets were plated (in triplicate) in a single well of 96-well plate containing either 250 µl Krebs–Ringer bicarbonate buffer/2.8 mM glucose or 250 µl Krebs–Ringer bicarbonate buffer/16.0 mM glucose and were incubated for 1 h at 37°C with 5% CO_2_. The supernatant was collected and stored at −80°C for insulin measurement.

### Cell proliferation assay, histology and immunostaining

To assay beta cell proliferation, BrdU was given to mice in drinking water (1 mg/ml) for 7 days. For histological examination, tissues were fixed overnight in buffered 4% para formaldehyde, embedded in paraffin and sectioned (4 µm thickness). Sections were mounted on positively charged slides (Fisher Scientific) and were stained with either hematoxylin-eosin or subjected to immunostaining with anti-insulin (R&D System) and anti-BrdU (BD Biosciences) antibodies. Primary antibody was detected by cy3 or FITC-conjugated antibodies.

Pancreatic and islet areas were measured from pancreatic sections from four mice each of the wild type and mutant genotypes using Axioskop (Axivision software). Islet mass per pancreas was calculated by multiplying relative islet area with wet mass of pancreas.

### Adenovirus generation and cell proliferation assay

To overexpress WDR13 protein in various cell lines, *Wdr13* adenovirus constructs were generated. In brief, *Wdr13* cDNA was amplified from pCMV-FLAG-*Wdr13* vector using T7 forward primer and reverse primer 5′GCTCTAGAGCAGCACAGGGTGACAGAACC3′, digested with *Pme*I and cloned at *Eco*RV of pAdTrack-CMV vector. AdGFP or Ad*Wdr13* were generated in HEK293T packaging cell line according to He et al., [47]. 10,000 MIN6 cells (obtained from National Centre for Cell Science, Pune, India) were seeded per well of 24 well plates [48] in DMEM media containing 10% FBS and were transfected either with AdGFP or Ad*Wdr13* using a titer of 100 MOI. Cell number was monitored by MTT assay at 24 h interval. For western blot analysis, MIN6 cells were transfected with AdGFP or Ad*Wdr13* virus (100 MOI) and the cells were lysed 48 hour post transfection.

### siRNA knockdown of WDR13 in MIN6 cells

MIN6 cells were transfected with non-specific scrambled (Scr) siRNA (Sc-37007) and *Wdr13*-specific siRNA (Sc-155258-Santacruz) using lipofectamin TM2000 (Invitrogen) in 30 mm dish (100 pmole each). The cells were lysed after 72 h post transfection and analyzed by immunoblotting.

### Chromatin immunoprecipitation

MIN6 cells were transfected with AdGFP and Ad*Wdr13* using 20 MOI and allowed to grow for 48 hours. The cells were crosslinked in 1% formaldehyde for 10 minutes at room temperature and scraped in PBS containing protease inhibitors. Chromatin immunoprecipitation was performed using the chromatin immunoprecipitation (ChIP) Assay Kit, Millipore (Catalogue Number: 17–295) as per the manufacturer's instructions. Briefly, cell lysate was precleaned by incubating in agarose beads for 1 h at room temperature followed by immunoprecipitation with anti-FLAG agarose beads for 3 h. The beads were washed and the genomic DNA fragments were eluted for identification of various promoter regions by PCR (Table S3). The experiments were repeated two times.

### Statistical analysis

The unpaired two-tailed *t* test was used for statistical analysis. Microsoft Excel software was used for calculation of *P* values. A *P* value<0.05 was considered significant. Data are presented as mean ± SEM.

## Supporting Information

Figure S1 **Feed consumption (gram/day) by** ***Wdr13*** **knockout mice and their wild type littermates.**
(TIF)

Table S1 **Effect of** ***Wdr13*** **genotype on litter size.**
(DOC)

Table S2 **Weight (in grams) of various organs from** ***Wdr13*** **mutant and their wild type littermates at 12 months.**
(DOC)

Table S3 **List of primers.**
(DOC)
