# Supplementary figures and images for: Lack of Wdr13 Gene in Mice Leads to Enhanced Pancreatic Beta Cell Proliferation, Hyperinsulinemia and Mild Obesity
Source: PLoS One. 2012 Jun 8;7(6):e38685. doi: 10.1371/journal.pone.0038685 (PMC3371019; doi:10.1371/journal.pone.0038685)

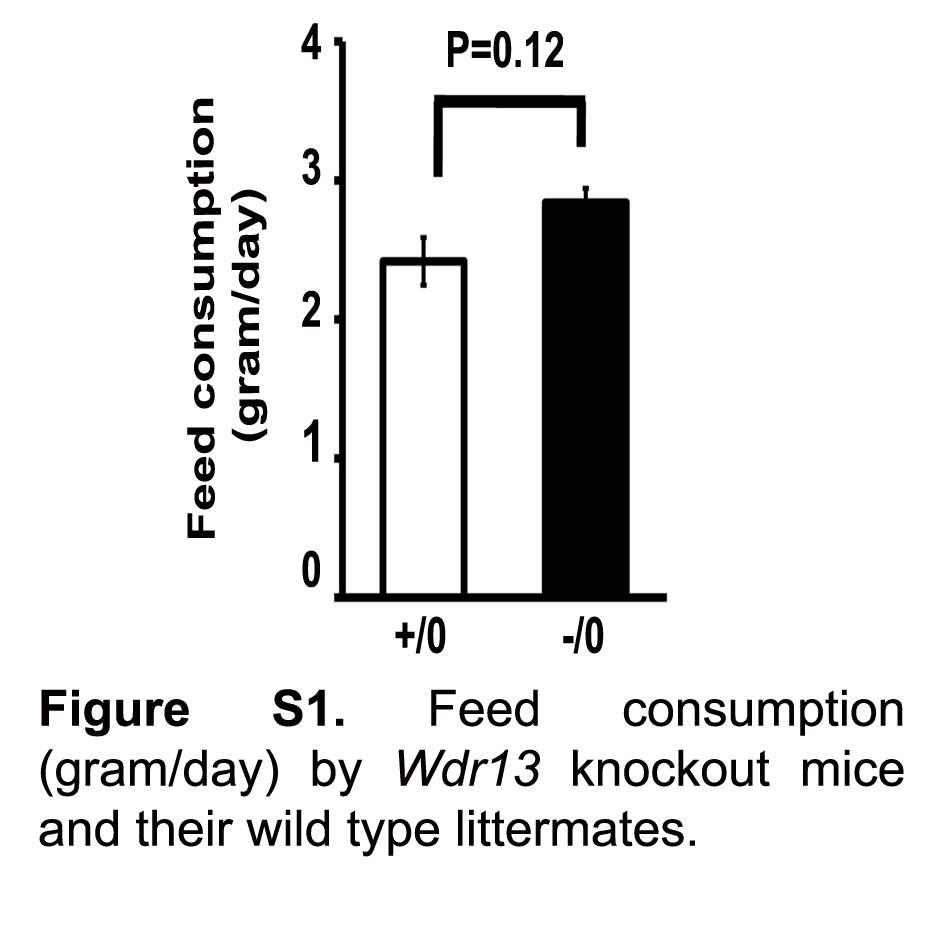

Supplement: Figure S1 — Feed consumption (gram/day) by Wdr13 knockout mice and their wild type littermates. (TIF) [file pone.0038685.s001.tif]
