# Supplementary material for: Lack of Wdr13 Gene in Mice Leads to Enhanced Pancreatic Beta Cell Proliferation, Hyperinsulinemia and Mild Obesity
Source: PLoS One. 2012 Jun 8;7(6):e38685. doi: 10.1371/journal.pone.0038685 (PMC3371019; doi:10.1371/journal.pone.0038685)
Supplement: Table S1 — Effect of Wdr13 genotype on litter size. (DOC) [file pone.0038685.s002.doc]

**Table S1- Effect of *Wdr13* genotype on litter size**

| **Mating type** | **Number of matings** | **Average litter size­** |
| --- | --- | --- |
| *Wdr13* -/0 Χ *Wdr13* -/+ | 6 | 11.7 |
| *Wdr13* +/0 Χ *Wdr13* -/+ | 7 | 10.6 |
| *Wdr13* -/0 Χ *Wdr13* +/+ | 5 | 10.4 |
| *Wdr13* -/0 Χ *Wdr13* -/- | 6 | 09.8 |
