# Supplementary material for: Lack of Wdr13 Gene in Mice Leads to Enhanced Pancreatic Beta Cell Proliferation, Hyperinsulinemia and Mild Obesity
Source: PLoS One. 2012 Jun 8;7(6):e38685. doi: 10.1371/journal.pone.0038685 (PMC3371019; doi:10.1371/journal.pone.0038685)
Supplement: Table S2 — Weight (in grams) of various organs from Wdr13 mutant and their wild type littermates at 12 months. (DOC) [file pone.0038685.s003.doc]

**Table S2- Weight (in grams) of various organs from *Wdr13* mutant and their wild type littermates at 12 months**

| **Genotype/ Tissues** | **Pancreases** | **Testis / Uterus** | **Liver** | **Brain** | **Heart** | **Kidney** | **Spleen** | **Lung** |
| --- | --- | --- | --- | --- | --- | --- | --- | --- |
| ***Wdr13* +/0 (n=8)** | 0.22±0.01 | 0.18±0.01 | 1.67±0.05 | 0.48±0.01 | 0.20±0.01 | 0.58±0.02 | 0.06±0.005 | 0.25±0.01 |
| ***Wdr13* -/0 (n=8)** | 0.30±0.01* | 0.18±0.01 | 1.85±0.10 | 0.49±0.01 | 0.21±0.01 | 0.62±0.02 | 0.08±0.004 | 0.27±0.01 |
| ***Wdr13* +/+ (n=6)** | 0.33±0.02 | 0.87±0.29 | 1.46±0.07 | 0.49±0.01 | 0.18±0.01 | 0.41±0.01 | 0.11±0.008 | 0.22±0.01 |
| ***Wdr13* -/- (n=6)** | 0.41±0.06* | 0.63±0.12 | 1.50±0.07 | 0.52±0.01 | 0.18±0.01 | 0.45±0.03 | 0.12±0.007 | 0.31±0.11 |

* P ≤ 0.05
