# Supplementary material for: Lack of Wdr13 Gene in Mice Leads to Enhanced Pancreatic Beta Cell Proliferation, Hyperinsulinemia and Mild Obesity
Source: PLoS One. 2012 Jun 8;7(6):e38685. doi: 10.1371/journal.pone.0038685 (PMC3371019; doi:10.1371/journal.pone.0038685)
Supplement: Table S3 — List of primers. (DOC) [file pone.0038685.s004.doc]

**Table S3 - List of primers**

| S.N. | Primer name | Primer sequences 5’-3’ |
| --- | --- | --- |
| 1 | *Wdr*13E2F  *Wdr*13E2R | AACGCCTACCGTACACCAAC  TGCTATAGGCACGAGCACTG |
| 2 | NeoF  NeoR | GATCGGCCATTGAACAAGAT  ATACTTTCTCGGCAGGAGCA |
| 3 | GAPDHF  GAPDHR | ACCCAGAAGACTGTGGATGG  CACATTGGGGGTAGGAACAC |
| 4 | p21CHIPF  p21CHIPR | CAGGCTGGTCTTGAACCTGT  AGGCATTCAAGGTCGTTTTG |
| 5 | CHIP CON F  CHIP CON R | TGGAACTGCTTCTGGTGAAC  ATCCGCCTCTGGCATTTTGG |
